# Diffuse Arterial Atherosclerosis Presenting With Acute Ischemic Gastritis

**DOI:** 10.7759/cureus.29115

**Published:** 2022-09-13

**Authors:** Mohamed Osman, Abu-Bekr Mohamed, Ahmed Salim

**Affiliations:** 1 Internal Medicine, Mayo Clinic, La Crosse, USA; 2 Internal Medicine, Detroit Medical Center, Wayne State University, Detroit, USA; 3 Internal Medicine, Interfaith Medical Center, New York City, USA

**Keywords:** intracardiac clot, embolic atherosclerotic plaque, embolic disease, diffuse atherosclerosis, ischemic gastritis

## Abstract

Ischemic gastritis is a rare cause of gastrointestinal bleeding due to the rich blood supply of the stomach. A 66-year-old lady with a history of coronary artery disease and hyperlipidemia presented with acute abdominal pain, diarrhea, and recent coffee-ground emesis. She had diffuse abdominal tenderness on physical exam with mild leukocytosis. A CT scan of the abdomen showed new peripheral wedge-shaped hypoenhancing regions in the spleen and both kidneys, suggestive of embolic infarcts. Posterior pneumatosis of the stomach suggestive of gastric wall ischemia was detected, with associated portal venous gas. She was started on heparin anticoagulation, broad-spectrum antibiotics, and intravenous fluids and underwent an urgent upper endoscopy showing hemorrhagic mucosa and fibrinous debris. A transesophageal echocardiogram showed mild aortic atherosclerosis. A CT angiogram of the abdomen showed calcified plaques at the origins of the hepatic artery, celiac artery, and superior mesenteric artery with severe ostial narrowing. Symptoms resolved, and she was started on statin therapy, prophylactic anticoagulation with apixaban, and high-dose peptic ulcer prophylaxis with pantoprazole. This ischemic gastritis case with multiple embolic spleen and kidney lesions is likely a sequela of extensive atherosclerosis and suggests that extensive atherosclerosis should be considered as a rare cause of acute gastrointestinal bleeding.

## Introduction

Ischemic gastritis is a rare cause of gastrointestinal bleeding as the stomach has an extensive blood supply with multiple vascular collaterals. Post-interventional embolization, hypotension, hemodynamic disturbances in the presence of celiac axis stenosis, vasculitis, and idiopathic causes were reported as causes of ischemic gastritis in a multicenter case series study [1]. It was also observed following acute gastric distension [2]. It can present with abdominal pain, vomiting, fever, and bleeding. Its diagnosis entails combining a constellation of radiological findings and endoscopic findings. Thumbprinting of the gastric contour and undulations are common radiological findings [3]. The development of intramural gas in ischemic gastritis due to gas-forming bacterial superinfection from the damaged mucosal surface or hematogenous spread is consistent with emphysematous gastritis and carries higher mortality [4-6]. Furthermore, the development of thickened gastric folds on imaging was noted [7].

On the other hand, acute mesenteric ischemia usually presents with acute severe abdominal pain, with a relatively normal physical examination. Acute mesenteric ischemia can be induced by arterial thrombosis, arterial embolism, non-occlusive mesenteric ischemia, and mesenteric venous thrombosis and can lead to the development of intestinal gangrene [8]. Chronic mesenteric ischemia presents with severe postprandial abdominal pain before reaching a plateau and resolving in two hours. It was found to be associated with *Helicobacter pylori*-negative gastric ulcers that resolved with revascularization [9,10].

Conservative therapy with intravenous fluids, antibiotics, intravenous proton pump therapy, and gastric decompression might lead to the complete resolution of ischemic gastritis [11]. Angiographic intervention is indicated in splanchnic vascular obstruction, while surgical intervention is indicated in cases of failure of conservative medical therapy, gastric perforation, gastric volvulus, and the development of gastric gangrene [12-14]. We present a rare case of diffuse arterial atherosclerosis presenting with acute ischemic gastritis.

## Case presentation

A 66-year-old female presented with a three-day history of abdominal pain and diarrhea. She had nausea with associated coffee ground vomiting one day prior to admission. She also reported mild postprandial abdominal pain for one month before admission resulting in a reduced oral intake.

Of note, the patient had a known history of coronary artery disease with previously placed cardiac stents, for which she was on aspirin 81 mg daily, antiplatelet therapy, dyslipidemia, gastroesophageal reflux disease (GERD), diabetes mellitus, chronic heart failure, and hypertension. She had no known history of atrial fibrillation, and cardiac monitoring showed a normal sinus rhythm on admission and throughout hospitalization.

On presentation, physical examination showed diffuse abdominal tenderness. Complete blood count showed a normal hemoglobin level of 12.6 g/dl, mild leukocytosis with a white blood cell count of 12500/l with neutrophilic predominance, and a normal platelet count of 176000/l. She had a normal kidney function with a serum creatinine level of 0.72 mg/dl and a normal blood urea nitrogen level of 11 mg/dl. Serum lactate level was normal at 1.1 mmol/l. She had a normal sodium level of 137 mmol/l and mild hypokalemia with a potassium level of 3.2 mmol/l. Hepatic function panel showed a normal total bilirubin level of 0.7 mg/dl, a normal direct bilirubin level of <0.2 mg/dl, a normal aspartate aminotransferase level of 29 u/l, a normal alanine aminotransferase level of 25 u/l, a slightly increased alkaline phosphatase level of 112 u/l, a normal albumin level of 4.1 g/dl, and a normal total protein level of 7.6 g/dl. Prothrombin time was 14 seconds with an international normalized ratio (INR) of 1.3, and activated partial thromboplastin time was normal at 27 seconds. Urinalysis showed no evidence of leukocytosis, proteinuria, or hematuria. A nasogastric tube was placed at the emergency department, which drained coffee-ground gastric secretions.

**Figure 1:**
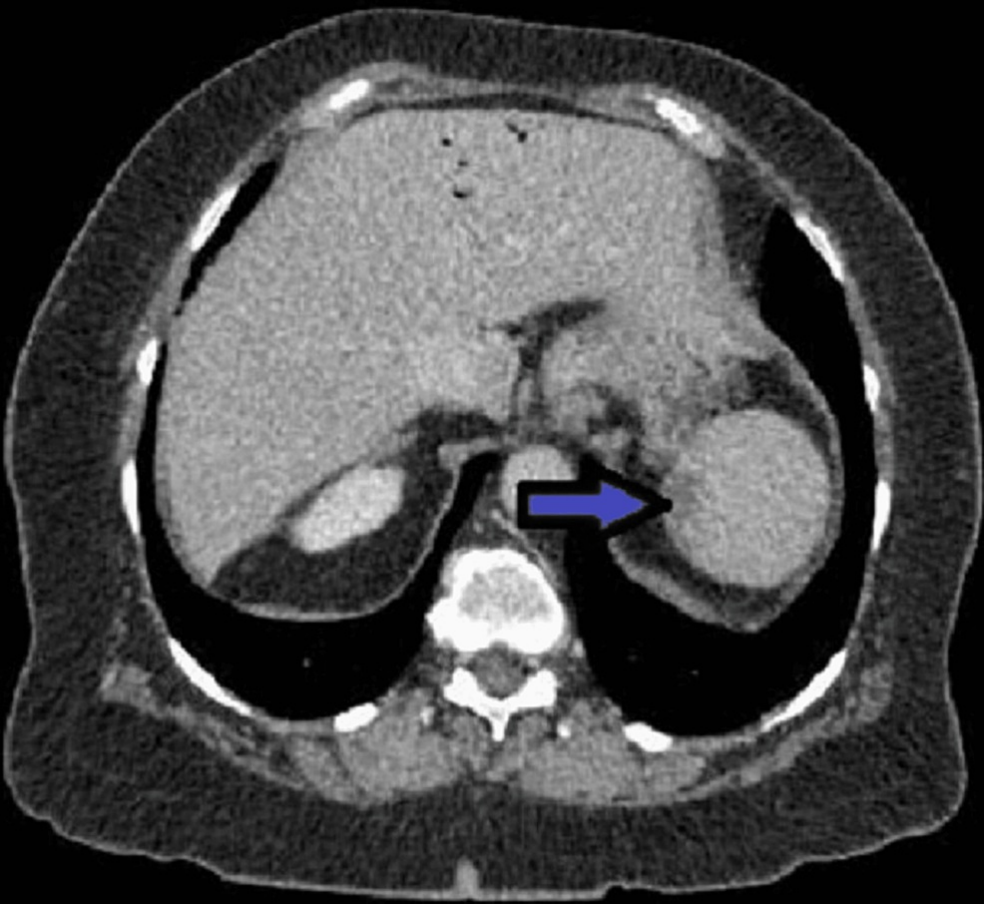
Hypoenhancing lesions in the spleen (arrow)

**Figure 2:**
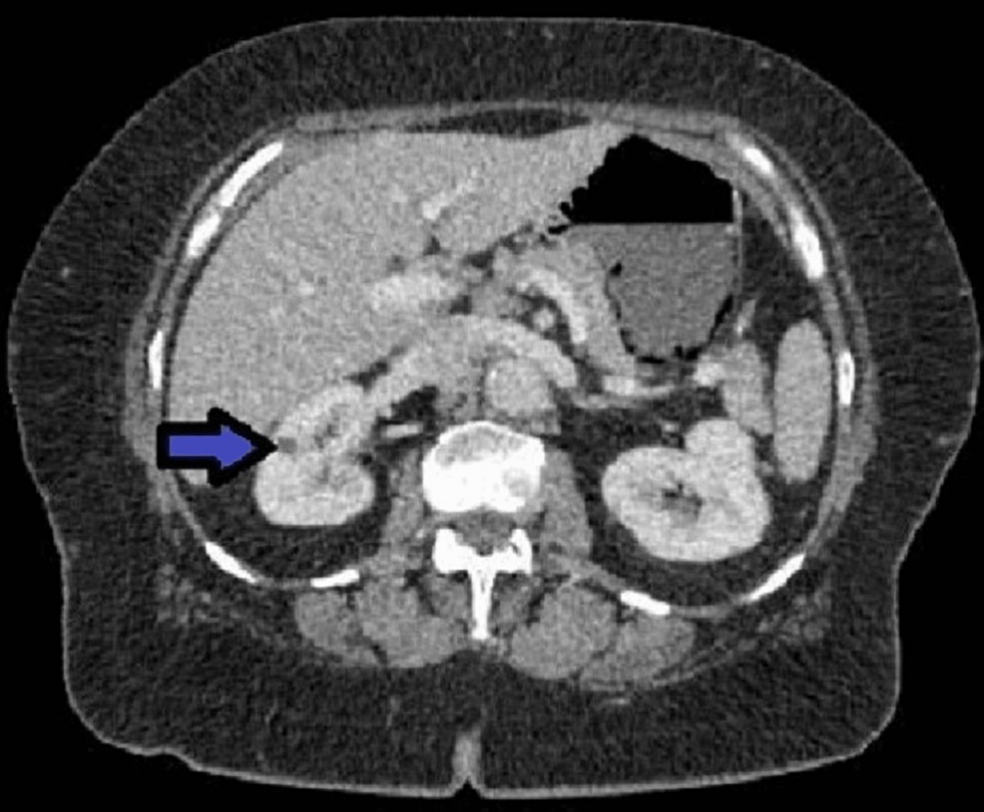
Hypoenhancing lesions in the kidneys (arrow)

**Figure 3:**
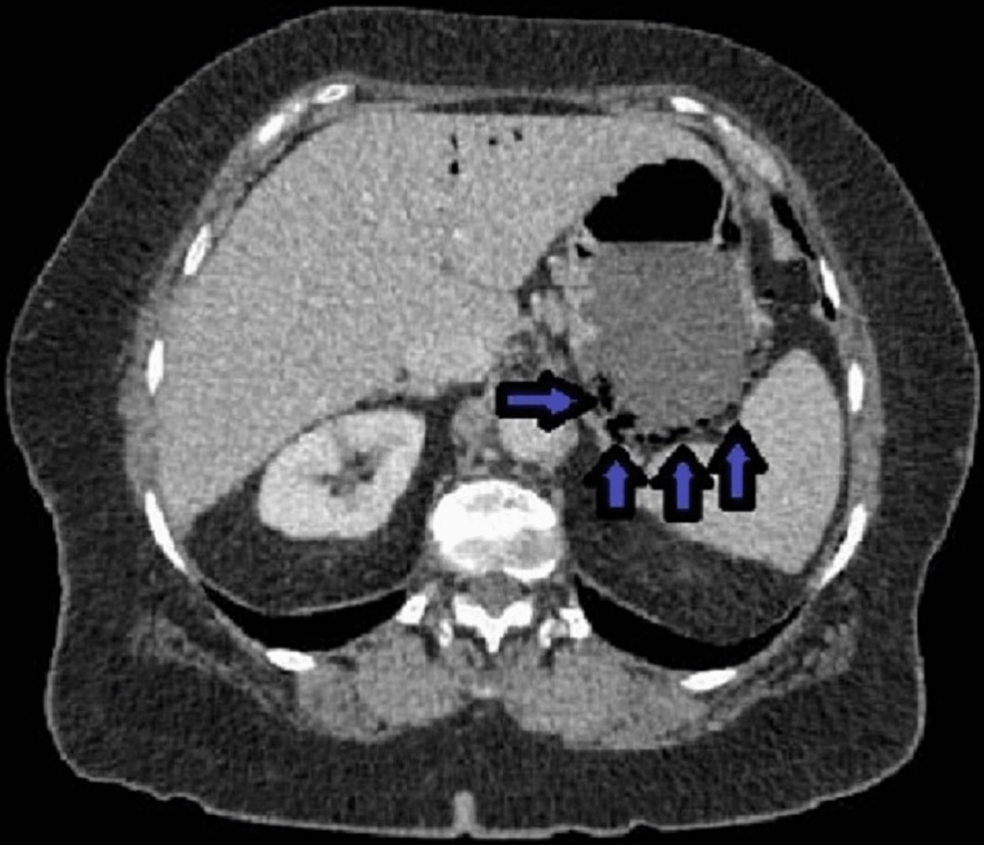
Posterior gastric wall pneumatosis (arrows)

**Figure 4:**
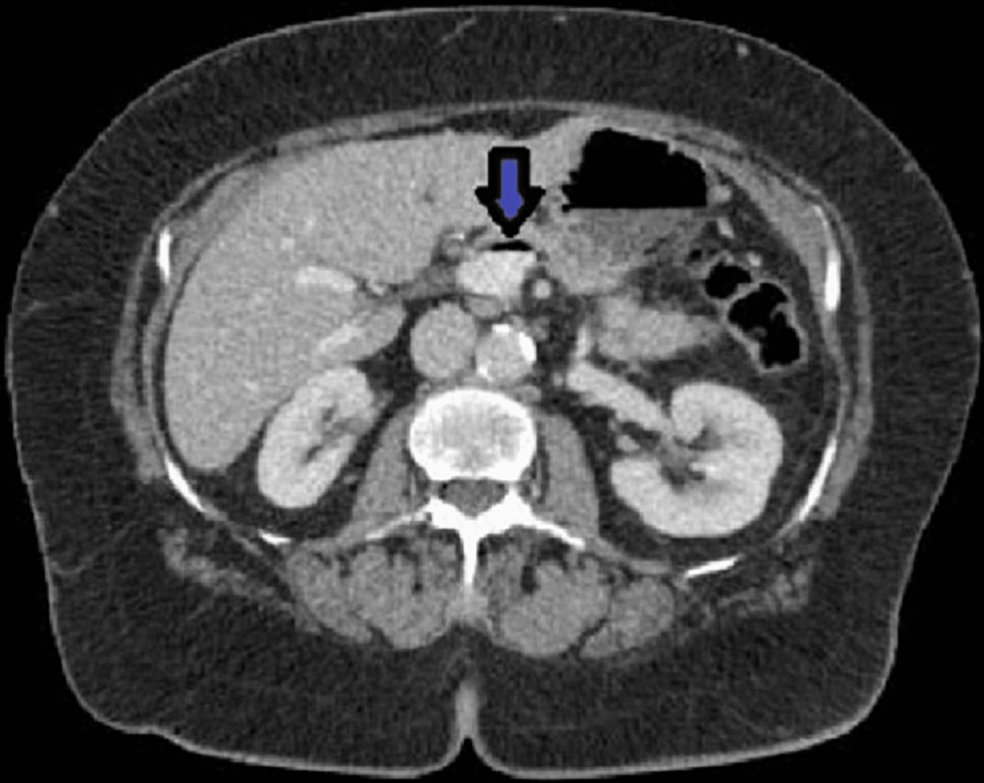
Portal venous gas (arrow)

A CT scan of the abdomen done on admission showed new small peripheral wedge-shaped hypo-enhancing regions in the spleen (Figure 1) and both kidneys (Figure 2), suggestive of infarcts from an embolic source. In addition, there was a large area of pneumatosis involving the posterior wall of the stomach (Figure 3) with associated portal venous gas (Figure 4). These findings were suggestive of an embolic phenomenon causing acute gastric wall ischemia.

She was commenced on broad-spectrum intravenous antibiotics, metronidazole 500 mg every eight hours and ceftriaxone 2 grams daily, intravenous fluids with 1000 ml bolus of normal saline, and intravenous proton pump inhibitor therapy on pantoprazole 40 mg oral every 12 hours. An initial 80 units/kg bolus of heparin anticoagulation was started, followed by an initial continuous infusion of 18 units/kg/hour, with follow-up rates adjusted to maintain the anticoagulation target based on institutional protocol. She was continued on antibiotics and maintenance fluids. She underwent urgent upper GI endoscopy, which showed a hemorrhagic-appearing mucosa on the anterior aspect of the stomach. The posterior aspect of the stomach was covered with fibrinous debris. Upon irrigation of the fibrinous debris, a pink and hemorrhagic mucosa was found. Similar findings were found in the distal stomach. There was no sign of frank necrosis. No biopsies were taken. Duodenum was not scoped. 

A transthoracic echocardiogram revealed a normal ejection fraction of 61%, with no wall motion changes, visible intracardiac mass, or thrombus. This was followed by a transesophageal echocardiogram which showed mild immobile atherosclerosis of the aortic arch and descending thoracic aorta without evidence of intracardiac thrombus, tumor, valvular vegetations, or spontaneous atrial contrast.

**Figure 5:**
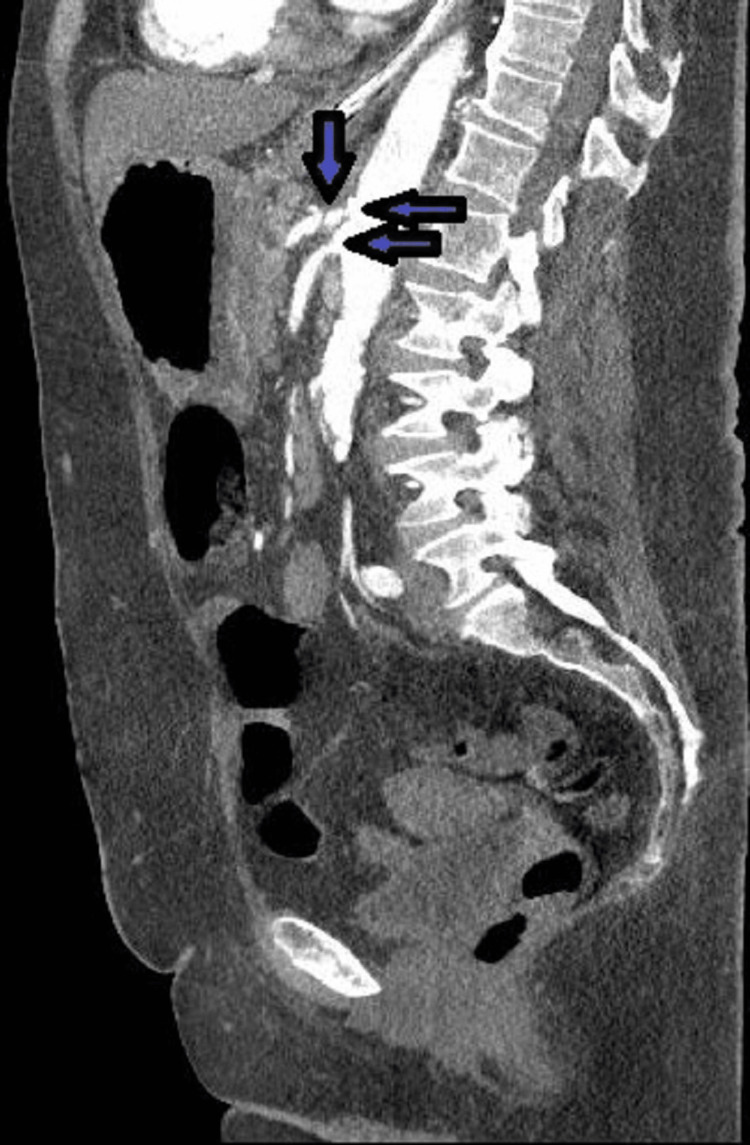
Calcified plaques at the origins of the celiac artery and the superior mesenteric artery (arrows)

CT angiograms of the chest, abdomen, and pelvis were done on the following day of admission showing the common hepatic artery arising directly from the aorta. Calcified plaques at the origins of the hepatic artery, celiac artery, and superior mesenteric artery were identified with severe ostial stenosis (Figure 5). In addition, there was high-grade stenosis at the origin of the right renal artery. There was a patent left renal artery with minimal stenosis. There was a patent inferior mesenteric artery with a calcified plaque at the origin, causing moderate stenosis. There was a calcified plaque causing moderate stenosis of the proximal right common iliac artery as well as moderate stenosis at the origin of the left internal iliac artery. Portal venous system gas was nearly resolved, while pneumatosis involving the gastric wall was completely resolved at this point, one day after treatment was started.

Blood cultures yielded no growth. Lipid panel was within normal limits, the low-density lipoprotein (LDL) level was 81 mg/dl, and the triglyceride level was 105 mg/dl. Antiphospholipid syndrome, malignancy, myeloproliferative disorder, paroxysmal nocturnal hemoglobinuria, vasculitis, and hereditary thrombophilia screening came back negative. Particularly, anti-cardiolipin IgG and IgM antibodies came back negative at less than <9.4 mpl, beta-2 glycoprotein 1 IgM and IgG antibodies came back negative at <9.4 u/ml, and dilute Russell viper venom time (DRVVT) ratio came back negative at 0.97 excluding antiphospholipid syndrome. Antineutrophil cytoplasmic antibodies (ANCA) panel came back negative at <0.2 U, antinuclear antibody (ANA) was mildly elevated at 4.6 U, creatine kinase (CK) level was normal at 69 u/l, and cryoglobulin level was negative. Protein electrophoresis was negative, and free light chain levels showed kappa of 2.1 mg/dl and lambda of 1.7 mg/dl. Paroxysmal nocturnal hemoglobinuria (PNH) phosphatidylinositol (PI)-linked antigen and Janus kinase 2 (JAK2) mutation testing came back negative. ADAMTS-13 activity came back normal at >100%, homocysteine level came back normal at 12.9 nmol/ml, while total serum homocysteine level was normal at 12.9 nmol/ml. Protein C activity was normal at 142%, free protein S antigen was normal at 108%, and antithrombin activity was normal at 90%. Since there was no evidence of resistance to activated protein C, no DNA-based testing for factor V Leiden disease was performed.

A brain MRI ruled out acute embolic stroke while showing moderate chronic microvascular ischemic changes, including a small chronic infarct in the left frontal operculum, raising the possibility of a previous embolic infarct.

Over the following two days, her abdominal pain gradually improved. Feeding was slowly introduced. Aspirin 81 mg daily home medication was discontinued, and the patient was discharged on oral anticoagulation on apixaban 5 mg every 12 hours, oral peptic ulcer prophylaxis on pantoprazole 40 mg every 12 hours for one month, and rosuvastatin on a lower dose of 5 mg daily due to a previous history of statin intolerance.

Two months after discharge, a repeat CT abdomen and pelvis angiogram was done for follow-up and showed similar-appearing high-grade stenosis at the origins of the celiac and hepatic arteries. The superior mesenteric artery was patent with less than 50% stenosis at the origin. Interval resolution of portal venous gas was noted. No ascites or dilated bowel loops were identified. 

## Discussion

This acute ischemic gastritis case with multiple embolic spleen and kidney lesions is likely the sequela of extensive atherosclerosis noted in different arterial segments, including the aorta. It suggests that extensive atherosclerosis should be considered as a cause of acute gastrointestinal bleeding due to ischemic gastritis, especially when aortic atherosclerosis is present. The patient had a known history of coronary artery disease with multiple cardiac stents placed in the past, which suggests an increased risk of atherosclerosis in other arteries. She was noted to have calcified plaques with severe ostial stenosis at the origins of the hepatic artery, celiac artery, and superior mesenteric artery, as well as severe ostial stenosis of the right renal artery and mild stenosis of the left renal artery suggesting that atherosclerosis was the likely cause of her ischemic gastritis. Although superior mesenteric artery stenosis was also identified in this patient, acute mesenteric ischemia is unlikely to be the cause of her symptoms since it does not explain the development of ischemic gastritis, the embolic lesions noted in the spleen and kidneys, and the normal radiological findings of the small and large intestine. The incidental finding of embolic lesions in both of her kidneys and the spleen is likely caused by dislodged atheroembolic plaques from the aorta or its branches that may have migrated distally through severely narrowed and diffusely atherosclerotic arteries. The previous small chronic frontal infarct noted on her brain MRI supports this possibility further. The absence of intracardiac clotting on transesophageal echocardiogram and atrial fibrillation on cardiac monitoring suggests that undetected arrhythmia like paroxysmal atrial fibrillation is less likely to be the cause of her ischemic gastritis, although still a possibility. 

Ischemic gastritis is a very rare disease entity and is under-reported in the medical literature except for a few reported case reports and case series [11]. There are no previous studies to our best knowledge that assessed its prevalence in the community. The largest retrospective case series study of ischemic gastritis, performed between January 2000 and May 2016 in a single, center identified only 17 patients with isolated gastric ischemia [15]. Another large multicenter case series study identified a total of 12 patients with ischemic gastritis, which was found to be caused by interventional radiological embolization procedures, hemodynamic changes in the presence of celiac axis stenosis, systemic hypotension, vasculitis, and other unidentified causes. Thirty-day and one-year mortalities were reported to be 33% and 41%, respectively, in this study [1].

Conservative therapy is the first line of therapy and might lead to the complete resolution of ischemic gastritis, including cases complicated by the development of emphysematous gastritis [16,17]. Acute gastrointestinal bleeding due to ischemic gastritis as a result of celiac artery stenosis induced by atherosclerosis can be successfully treated with celiac artery stenting [18]. Surgical intervention is indicated in cases unresponsive to conservative medical therapy or those complicated by gastric perforation, gastric volvulus, and the development of gastric gangrene that might require partial gastrectomy [12-14].

## Conclusions

Acute ischemic gastritis should be considered as the etiologic factor for acute gastrointestinal bleeding when diffuse arterial atherosclerosis and systemic embolism are detected, with or without a history of coronary artery disease or paroxysmal atrial fibrillation. Management of the condition involves conservative therapy with fluid resuscitation, broad-spectrum antibiotics, gastric decompression, and intravenous proton pump therapy. Anticoagulation should be considered in selective cases, especially when atrial fibrillation is suspected. Conservative therapy might lead to the complete resolution of ischemic gastritis cases, including those resulting in emphysematous gastritis. Angiographic intervention is reserved for celiac artery stenosis due to atherosclerosis, while surgical intervention is reserved for selective cases, including those complicated by gangrene of the gastric mucosa.
